# Optimization of Polysaccharide Hydrocolloid for the Development of Bioink with High Printability/Biocompatibility for Coextrusion 3D Bioprinting

**DOI:** 10.3390/polym13111773

**Published:** 2021-05-28

**Authors:** Wonseop Lim, Seon Young Shin, Jae Min Cha, Hojae Bae

**Affiliations:** 1 KU Convergence Science and Technology Institute, Department of Stem Cell and Regenerative Biotechnology, Konkuk University, Seoul 05029, Korea; seopp322@gmail.com (W.L.); tempast606@naver.com (S.Y.S.); 2 Department of Mechatronics Engineering, College of Engineering, Incheon National University, Incheon 22012, Korea; j.cha@inu.ac.kr; 3 3D Stem Cell Bioengineering Laboratory, Research Institute for Engineering and Technology, Incheon National University, Incheon 22012, Korea

**Keywords:** 3D bioprinting, printability, biocompatibility, pore size, carboxy methyl cellulose, xanthan gum

## Abstract

Bioink is the main component of 3D bioprinting process and is crucial for the generation of sophisticated 3D structures through precise spatial control. Therefore, bioink’s core material must have characteristics that support good printability as well as biocompatibility. However, there is a lack of bioinks developed that satisfy these characteristics at the same time. In this work, our aim was to develop a bioink that satisfies the needs for both printability and biocompatibility through effectively utilizing hydrocolloid materials. To do so, carboxymethyl cellulose (CMC) and xanthan gum (XG) were used to maintain proper shear properties at high pressure and increase the mechanical properties of bioink without excessively affecting the viscosity, and thus enhance printability and biocompatibility. Various bioink formulations were applied to 3D printing process and the printability optimization was carried out through adjusting the hydrocolloid contents in connection with different cross-linking methods. Through utilization of hydrocolloids, the printability and rheological analysis showed that the bioink has improved mechanical properties and confirmed that the printability could be adjusted by controlling the CMC and XG ratio. Moreover, cell viability and immunocytochemical staining analyses showed cell compatibility with enhanced stability. The proposed convenient method to control the printability with improved biocompatibility suggests more appropriate use of bioink for co-axial 3D bioprinting.

## 1. Introduction

3D bioprinting is a highly versatile technology that can be used to realize the dynamic hierarchical architecture of tissues and/or organs based on biomaterials and living cells to address the significant demands for numbers of the clinical fields such as customized tissue/organ transplantation surgery [1]. Depending on the technical features of printing process, 3D bioprinting technologies can be classified into three approaches such as inkjet-based printing, laser-based printing, and extrusion-based printing for which suitable printing materials, such as cells and bioinks, are customized according to the purpose of printing. The most important task for the successful 3D bioprinting would be to choose or develop the desirable bioink that can reflect the biochemical and physical microenvironment of the targeted tissue to print (biocompatibility) [2,3,4,5,6,7]. In addition, the chosen bioink should meet the printing processability to enable the precise construction of 3D tissue through layer-by-layer depositions with the specific measurements given to the printer (printability). Such bioinks also need to have appropriate rheological properties to secure the cell viability from mechanical stress, such as shear stress, which could be generated by nozzle-based printing procedures [8,9,10]. Previously, the printability of bioink has been studied in relation with viscosity and rheological properties. For instance, during the 3D bioprinting process, appropriate viscosity is required for the bioink to maintain the acceptable level of mechanical strength to prevent the alteration of the printed structure [11]. Biocompatibility has been reported to rely on the porosity of hydrogel such that many of the cellular behaviors such as cell adhesion, migration, proliferation, differentiation, and secretion of extracellular matrix (ECM) are readily influenced by the availability of cell adhesive surface area [12,13,14,15,16,17].

**Figure 1:**
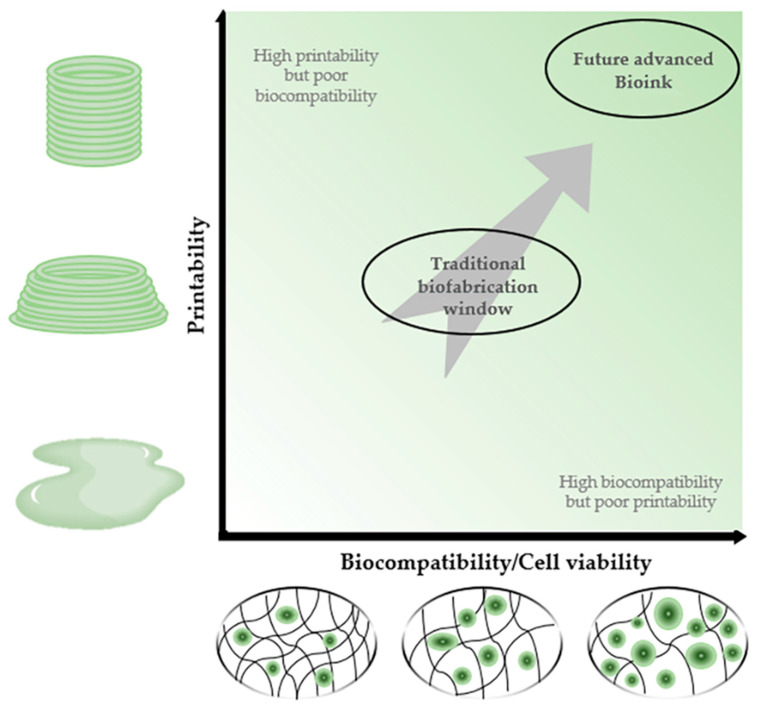
Description of the 3D bioprinting printability/biocompatibility window. Proposed bioink must have properties that satisfy both printability and biocompatibility unlike conventional one.

As mentioned above, bioink has been developed to address the challenges in both biocompatibility and printability together [18,19]. However, addressing one factor usually goes against the other such that low viscosity of bioinks could provide superior biocompatibility with high availability of cell adhesive surface area (namely high porosity), while its printability would not be sufficient to uphold the mechanical stability of the printed structure [20,21,22]. In other cases, the concentration of polymers has been elevated along with various solvents used for hydrogel synthesis to increase the printability and reinforce the mechanical strength of printed structure [23,24], which improperly influenced the biocompatibility [25]. Therefore, it is crucial for the newly developed bioinks to meet the fundamental requirements of both printability and biocompatibility [26,27] (Figure 1).

This study aims to develop a bioink that can satisfy both biocompatibility and printability all at once by employing the optimized composition of four different biomaterials, such as gelatin methacryloyl (GelMA), alginate, carboxymethyl cellulose (CMC), and xanthan gum (XG). GelMA is a hydrophilic light-responsive polymer produced by reacting methacrylic anhydride with gelatin and has an advantage of providing good cell adhesion along with tunable physicochemical properties [28]. In particular, GelMA-based bioink has been proven to show characteristics similar to natural ECM and has been widely used for 3D cell culture and tissue formation [29,30]. Alginate is an anionic polysaccharide extracted from seaweed and can form hydrogel by divalent cations-alginate complex formation, which has been proven to be biocompatible and suitable for the extrusion-based bioprinting [31,32]. Previous studies demonstrated that the lack of cell adhesion sites and controlled degradability of native alginate hydrogel could be improved by the alginate/GelMA composite hydrogel and successfully 3D-bioprinted as a bioink using co-axial types of nozzles equipped in an extrusion-based bioprinter [33,34,35]. Hydrocolloid has been used to regulate the shear properties of substances to form gels with the ability to modulate viscosity of hydrogels in a wide range [36]. CMC is a hydrophilic polysaccharide hydrocolloid with a high molecular weight that has been reported to show the advantages for maintaining desired shear properties of hydrogel at high pressure and high temperature conditions [37]. Additionally, it has been reported that cell adhesion and migration could be supported by incorporated proteins binding with CMC’s matrix [38]. XG is another hydrophilic polysaccharide hydrocolloid used as an effective viscosity regulator as supplemented to a hydrogel composition, which enables to modulate the viscosity even at the low concentration [39]. Furthermore, XG’s shear thinning property could contribute to increasing the mechanical properties of the resultant bioink prepolymer solution without excess influences on in situ viscosity of a hydrogel composition [40].

Here, using the abovementioned characteristics, CMC and XG were added in different concentration ratios (1:1, 1:2, 2:1, and 2:2) to further enhance the printability of bioink and thus took part as the backbone and supporting biomaterial. In addition to this, three different cross-linking methods (ion, UV, and ion + UV) have been applied to provide tunability regarding pore size and mechanical strength optimization.

## 2. Materials and Methods

### 2.1. Materials

Gelatin was obtained from cold water fish skin, methacrylic anhydride (MA, contains 2000 ppm Topanol A as an inhibitor, 94%), lithium phenyl-2,4,6-trimethylbenzoylphosphinate (LAP), alginic acid sodium salt, calcium chloride dihydrate, and xanthan gum (XG) from *Xanthomonas campestris*, and polydimethylsiloxane (PDMS, Sylgard^®^) were purchased from Sigma-Aldrich (St. Louis, MO, USA). Dulbecco’s modified eagle’s medium (DMEM, high glucose), 1X Dulbecco’s phosphate buffered saline (DPBS), fetal bovine serum (FBS), 1X penicillin-streptomycin solution (P/S), and 1X trypsin-EDTA solution were purchased from Welgene (Gyeongsan, Gyongsangbuk-do, Korea). Carboxymethyl cellulose sodium salt (CMC) was purchased from Daejung (Siheung, Gyeonggi-do, Korea). Cell counting kit-8 (CCK-8) was purchased from Dojindo (Rockville, MD, USA). Live/Dead assay kit was purchased from Thermo Fisher Scientific (Waltham, MA, USA). Additionally, 4′,6-Diamidino-2-phenylindole, dihydrochloride (DAPI), and Alexa Fluor™ 488 and 594 were purchased from Invitrogen (Carlsbad, CA, USA). Anti-human nuclear antigen antibody (HNA), anti-Ki-67 antibody, and anti-matrix metallopeptidase-9 (MMP-9) antibody were purchased from Abcam (Tokyo, Japan).

### 2.2. High Printability Bioink Development

#### 2.2.1. Gelatin Methacryloyl (GelMA) Synthesis

After dissolving 10% (*w*/*v*) gelatin in 1X PBS at 50 °C for 30 min, 10% MA was added at a rate of 0.5 mL/1 min and reacted for 2 h while maintaining at 50 °C. To terminate the reaction, excess amount of 1X PBS was added (4 times the volume) to the reacted solution. To remove any unreacted methacrylic anhydride, dialysis was performed using 12–14 kDa dialysis membrane tube (Spectrum Laboratories, Rancho Dominguez, CA, USA), and fresh distilled water was replenished 2~3 times per day while maintaining at 40 °C. After the dialysis, final solution was filtered using a 1.2 μm membrane filter. The filtered solution was frozen at −80 °C for 1 day and lyophilized for 7 days to obtain GelMA.

#### 2.2.2. Preparation of Bioink

GelMA, alginate, CMC, and XG were used in combination for the bioink development. First, GelMA solution was prepared by dissolving 0.05% (*w*/*v*) LAP in 1X PBS at 80 °C, then lyophilized GelMA was dissolved at 37 °C for 1 day. Alginate solution was made by adding alginic acid sodium salt powder to 1X PBS at room temperature and stirred at a speed of 100 rpm for 1 day. Carboxymethyl cellulose sodium salt (CMC) solution was made by first completely dissolving CMC using glycerin (Duksan, Gyeonggi-do, Korea), and then same amount of 1X PBS was added. Xanthan gum (XG) solution was prepared using the same method as CMC solution.

The individually prepared solutions were mixed in the different ratios using a trial-and-error method to initially acquire the high printability/biocompatibility window using the co-axial bioprinting technique. For the optimization, commercially available needles of different diameters, ranging from 18 to 27 G, were used. During the printing process, unmodified alginate undergoes gelation in the presence of calcium ion (Ca^2+^) and GelMA through UV exposure. The constructs were then printed according to cross-linking conditions (ion, UV, and ion + UV). Meanwhile, to obtain a desired diameter of the continuously printed fiber, the different prepolymer discharge speed was tested. Finally, the internal nozzle size of 25 G and external nozzle size of 18 G with the flow rate of 22 μL/min was employed for the experiments. For the bioink prepolymer solution, GelMA and alginate were fixed at 10% (*w*/*v*) and 2% (*w*/*v*), respectively, and CMC and XG were used at the concentration of 1% or 2% (*w*/*v*) throughout the study.

### 2.3. Evaluation of Physical Properties of Bioink

#### 2.3.1. Printability Test

To evaluate the printability, the different bioink formulations were extruded using 22 G nozzle. Gelatin methacryloyl (GelMA) and alginate were used at a fixed concentration of 10% and 2% (*w*/*v*), respectively. In the case of CMC and XG, 1:1, 1:2, 2:1, and 2:2 concentration ratios were used. The viscosity of the bioink was adjusted by the CMC and XG contents, and the printability optimization was carried out. To quantitatively define the printability according to varying compositions and cross-linking conditions, printability (Pr) values were calculated following the equation previously defined by Ouyang et al. (2016) [23,41]. The circularity (C) value is closer to 1 as the printed shape is closer to circle. The Pr value has been calculated by substituting each measured perimeter (L) and area (A) values. The larger values of Pr correlates to greater degree of gelation of the bioink and vice versa with Pr value within the range of 0.9–1.1 demonstrate sound filament morphology and mechanical stability of printed construct.

$$Pr=\frac{\pi }{4}\cdot \frac{1}{C}=\frac{L^{2}}{16A}.$$

#### 2.3.2. Rheological Characterization Test

Rheological characterization was performed according to CMC and XG concentrations (*w*/*v*) to confirm the appropriate rheological properties of the bioink. The test was performed after stabilizing bioink on a rheometer at 37 °C for 10 min before the measurement. The recorded measurements were the complex viscosity by shear rate and the Storage (G′)/Loss (G′′) modulus by angular frequency. In the case of shear rate, the measurement was performed in the range of 1–100 (s^−1^) and the angular frequency was measured in the range of 1–100 (rad/s) with shear strain fixed at 1%.

#### 2.3.3. Scanning Electron Microscopy

To confirm the internal pore structure after cross-linking, cross-sectioned hydrogel specimens were analyzed via a scanning electron microscope [14]. To prepare test specimen, bioink prepolymer solution was pipetted into the round-shaped PDMS mold with a diameter of 8 mm and a height of 5 mm and cross-linked under three conditions (ion cross-linking, UV cross-linking, and ion cross-linking after UV cross-linking (ion + UV)) with GelMA, alginate, CMC, and XG concentrations fixed at 10%, 2%, 2%, and 2%, respectively. The GelMA/alginate/CMC/XG hydrogel was fabricated using the method described in our previous study [35]. Briefly, initial cross-linking was done using 0.3 M CaCl_2_ for alginate gelation. Calcium ion (Ca^2+^) was diffused into the prepolymer solution containing alginate, and ionic cross-linking took place as the Ca^2+^ ion replaced the sodium ion. Second, covalent cross-linking was carried out through UV irradiation at an intensity of 6.9 mW/cm^2^. The prepared hydrogel specimens were first frozen instantly by immersing in liquid nitrogen for 30 s followed by freezing at −80 °C for 1 day. The samples were then lyophilized and cut vertically before the observation. The cross-section of the vertically cut hydrogel was observed using a field-emission scanning electron microscope (FE-SEM, Hitachi S-4300 model).

#### 2.3.4. Young’s Modulus

For testing, Young’s modulus test, the hydrogels were prepared using the same method as described for scanning electron microscopy observation. Bioink formulation cross-linked under three different conditions (ion, UV, and ion + UV) were analyzed using CT3 Texture Analyzer (Brookfield Engineering Laboratory, Stoughton, MA, USA). The specimens were compressed with a rate of 0.05 mm/s using a 12.7 mm diameter probe. The strain was measured in a range of 5–15% strain.

#### 2.3.5. Swelling and Degradation Test

For swelling and degradation test, the hydrogels were prepared using the same method as described for scanning electron microscopy observation, but using the PDMS mold with different height (height of 2 mm). Bioink prepolymer solution was cross-linked under three conditions (ion, UV, and ion + UV). The prepared hydrogels were then conditioned in 1 mL DMEM (10% FBS, 1% P/S). After 24, 48, and 72 h, the liquid remaining on the hydrogel surface was removed, and the weight of the hydrogel was recorded. The swollen hydrogel was frozen at −80 °C and lyophilization was performed. The weight of the lyophilized hydrogel was recorded. The degrees of swelling and degradation were measured, respectively, by mass swelling ratio and percent mass remaining.

### 2.4. Bioink Biocompatibility Evaluation

#### 2.4.1. 3D Cell Encapsulation

To evaluate the biocompatibility of the bioink, patient’s bone marrow-derived mesenchymal stem cells (hMSCs) were used (Catholic university hospital, Seoul, Korea). Cells were cultured using media containing DMEM with 10% FBS, 1X P/S. Cells were encapsulated at the concentration of 2.5 × 10^6^ cells/mL in bioink prepolymer solution with a composition of 10% GelMA, 2% alginate, 2% CMC, and 2% XG. The bioink prepolymer solution containing hMSCs was cross-linked under three conditions (ion, UV, and ion + UV). For ion cross-linking, two slide coverslips (height: 300 μm) on 1.5% agarose with 0.3 M CaCl_2_ were used as a spacer, and 10 μL bioink prepolymer solution was pipetted, and the calcium ion was allowed to fully diffuse for alginate gelation (20 s). For UV cross-linking, two slide coverslips (height: 300 μm) on the PDMS mold were used as a spacer, and 10 μL of bioink prepolymer solution was pipetted followed by UV irradiation.

#### 2.4.2. Live/Dead Assay

The hMSCs-encapsulated bioink prepolymer solution was cross-linked under three conditions (ion, UV, and ion + UV) and then cultured for 24, 48, and 72 h. Hydrogels cultured for 24, 48, and 72 h under three conditions were stained using the Live/Dead Kit. The stained hydrogels were observed using Lionheart FX (BioTek, Winooski, VT, USA).

#### 2.4.3. Cell Proliferation Assay

To confirm the degree of proliferation of 3D-encapsulated hMSCs, CCK-8 and BrdU assays were performed. The degree of proliferation was compared at 24, 48, and 72 h according to the cross-linking conditions (ion, UV, and ion + UV). For the CCK-8 assay, the hMSCs-encapsulated hydrogel was treated with CCK-8 and reacted in an incubator for 2 h. Subsequently, the absorbance was measured at a wavelength of 450 nm using an Epoch Microplate Spectrophotometer (BioTek, Winooski, VT, USA). For the BrdU incorporation assay, hMSCs-encapsulated hydrogel was cultured using 24 well plate, then treated with the BrdU cell proliferation kit (Millipore, Burlington, MA, USA), and reacted in an incubator for 2 h. To detect BrdU incorporation, an anti-BrdU monoclonal antibody was treated for 1 h, and goat anti-mouse IgG antibody and peroxidase conjugate was treated for 30 min. The tetramethylbenzene (TMB) peroxidase substrate treatment reaction was stopped after the color change. Signal intensity was measured at 450/550 nm using an Epoch Microplate Spectrophotometer (BioTek, Winooski, VT, USA).

#### 2.4.4. ELISA Assay

ELISA assay was performed to measure the expression of MMP-9, an important enzyme that belongs to the zinc-metalloproteinases family involved in the degradation of the ECM [42]. After culturing hMSCs-encapsulated hydrogels (ion, UV, and ion + UV), the supernatant was collected after 72 h and measurement was conducted using Quantikine^®^ ELISA (R&D Systems, Minneapolis, MN, USA). The experiment was performed without protease inhibitor and media change during the culture, and the concentration was measured as ng/mL.

#### 2.4.5. Phalloidin/DAPI Staining

To confirm the proliferation of 3D-encapsulated hMSCs and the morphology of filamentous actin, phalloidin/DAPI staining assay was performed after culturing for 72 h. The bioink prepolymer solution was cross-linked under two conditions (UV and ion + UV). The hMSCs-encapsulated hydrogel was fixed for 40 min using a 4% paraformaldehyde solution. Then, for the permeabilization, the hydrogel was reacted with 100% methanol for 10 min at room temperature. After permeabilization, hydrogel was reacted 10 µg/mL phalloidin reagent in blocking buffer for 2 h. Cell nuclei were also stained with 1 µg/mL DAPI reagent with DPBS at room temperature for 5 min. Stained cells and filamentous actin were observed using Lionheart FX (BioTek, Winooski, VT, USA).

#### 2.4.6. Immunocytochemistry Staining

To confirm the properties of cells encapsulated in hydrogel under UV and ion + UV cross-linked conditions, an immunocytochemistry staining assay was performed. At first, to evaluate the decomposition of ECM, MMP-9/DAPI staining assay was performed. The hMSCs-encapsulated hydrogel was fixed for 40 min using a 4% paraformaldehyde solution and reacted with 100% methanol for 10 min at room temperature, and normal goat serum (Vector Labs, Burlingame, CA, USA) was used as a blocking solution. Then, hydrogel was reacted with anti-MMP-9 antibody overnight at 4 °C. The secondary antibody was attached using goat anti-rabbit Alexa Fluor™. Cell nuclei were counterstained with 1 µg/mL DAPI reagent.

Next, to confirm the proliferation of encapsulated hMSCs, Ki-67/HNA/DAPI staining assay was performed. The same methods were used for fixation and permeabilization as described above. After blocking step using normal goat serum, hMSCs encapsulated hydrogel was reacted with anti-Ki67 and anti-HNA antibody overnight at 4 °C. The secondary antibody was attached using goat anti-rabbit Alexa Fluor™ 488 against Ki-67 and goat anti-mouse IgG Alexa Fluor™ 594 against HNA. Cell nuclei were also counterstained with 1 µg/mL DAPI reagent. All staining specimens were observed using Lionheart FX (BioTek, Winooski, VT, USA) and analysis was performed with ImageJ software (NIH, Bethesda, MD, USA).

### 2.5. 3D Bioprinting of hMSCs

To assess the cell viability of the printed cell, 3D bioprinting was performed. As the bioink has a sufficient printability for maintaining printed structure, two cross-linking conditions (UV and ion + UV) were evaluated. The hMSCs were harvested and suspended in bioink prepolymer solution containing 10% GelMA, 2% alginate, 2% CMC, and 2% XG at a final cell concentration of 2.5 × 10^6^ cells/mL. The extrusion-based 3D printing system was set up with a coaxial nozzle (internal nozzle size of 25 G and external nozzle size of 18 G) with a flow rate of 22 µL/min using a microfluidic syringe pump. To produce a grid patterned structure by ion + UV cross-linking, bioink was extruded from internal nozzle and 0.3 M CaCl_2_ was extruded from external nozzle. Ionic cross-linking occurs immediately during the entire printing process. After printing, the printed structure was cross-linked with UV and cultured using DMEM (10% FBS, 1X P/S). For printed structure cross-linked with UV only, bioink was extruded using internal nozzle only. The printed structures were cultured for 24 h, and then, the cell viability according to cross-linking conditions were evaluated.

### 2.6. Statistical Analysis

Pore size, Young’s modulus, and mass swelling ratio data were analyzed using GraphPad Prism 8.0.2 (GraphPad Software, La Jolla, CA, USA). The rest were analyzed using SigmaPlot 10.0 (Systat Software, San Jose, CA, USA) and statistical analysis were performed using SPSS software (SPSS Inc., Chicago, IL, USA). All the data are presented as the mean ± standard deviation (SD) and comparison of the mean value among the groups was conducted through unpaired Student’s *t*-tests. The *p*-value was used in three ranges set to be less than 0.001 (***, ^+++^), 0.01 (**, ^++^), and 0.05 (*) and was considered to indicate statistical significance.

## 3. Results and Discussion

### 3.1. Printability and Rheological Properties

**Figure 2:**
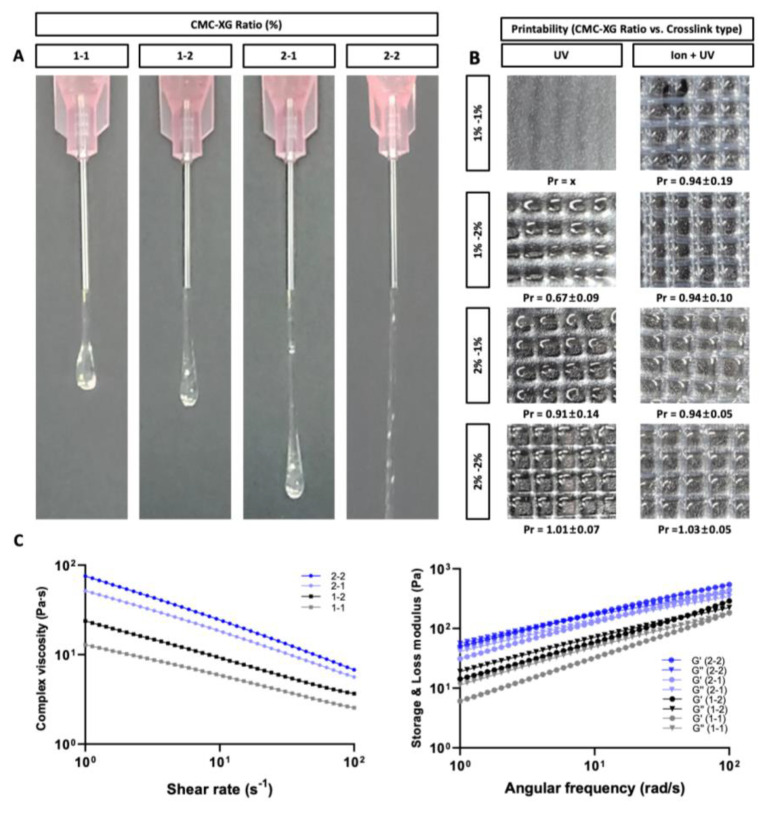
Printability optimization and rheological characteristics of different bioink formulations (CMC:XG concentration ratio set to 1:1, 1:2, 2:1, and 2:2). (**A**) Formation of fibers and droplets of bioink extruded from the nozzle. (**B**) Grid pattern of printed structure with calculated Pr value through the square shape in the grid pattern. Printability (Pr) value was calculated according to the equation defined by Ouyang et al. (2016) (*n* = 8) [23]. (**C**) Plot of complex viscosity by shear rate (left) and Storage (G′)/Loss (G′′) modulus by angular frequency.

The printability and stability of the printed object vary depending on the viscosity and rheological properties of the bioink. One method of intuitively measuring printability is to compare the fiber and droplet shapes of bioink printed through nozzles [43]. To confirm the printability, CMC:XG concentration ratio (*w*/*v* %) was set to 1:1, 1:2, 2:1, and 2:2, and a 20 G nozzle was used. A printability test was performed until the bioink was completely dispensed and the printing profile from the nozzle have been imaged (Figure 2A). In the case of bioink with 1:2 and 1:2 ratio, the bioink was printed under gelation behavior, which can be explained by droplet at the nozzle tip. However, the gelation behavior was tunable with the introduction of ionic cross-linking and thereby resulting in calculated Pr value of 0.94 ± 0.19 for 1:1 ratio and 0.94 ± 0.10 for 1:2 (Figure 2B). According to the previous study, Pr value within the range of 0.9–1.1 is considered good filament morphology and mechanical stability [23]. When the concentration of CMC was increased (2% *w*/*v*), the bioink was printed with a proper gelation state without the aid of ionic cross-linking forming the proper interconnected lattice construct resulting in a regular grid pattern. Correspondingly, the Pr value of 2:1 and 2:2 sample was 0.91 ± 0.14 and 1.01 ± 0.07, respectively. When ionic cross-linking is applied upon extrusion, the Pr value slightly increased for both 2:1 and 2:2 bioink sample indicating higher degree of gelation. Through this optimization study, it can be confirmed that the ideal printing condition can be established by modulating hydrocolloid concentrations and cross-linking method.

To further analyze the correlation between the rheological properties and the printability, the complex viscosity of the bioink along with the storage (G′) and loss (G′′) modulus were measured and confirmed (Figure 2C). According to the complex viscosity plot, it can be confirmed that the complex viscosity increased according to the CMC and XG concentrations. Furthermore, decreased complex viscosity was observed as the shear rate increased confirming shear-thinning property of the proposed bioink, which makes it suitable for increasing the mechanical properties of bioink prepolymer solution without affecting the viscosity [40]. In addition, the Storage (G′) and Loss (G′′) modulus for bioink prepolymer with different concentration ratios (1:1, 1:2, 2:1, and 2:2) of CMC:XG were observed. All the bioink formulation exhibited gel-like behavior as the G’ was higher than G’’ within the testing range. Further, bioink formulations showed liquid-like profiles as the angular frequency is increased (crossing point of G′ and G′′) indicating sol to gel phase transition, which results in reduction in the stress on bioink during the bioprinting process.

### 3.2. Pore Distribution and Young’s Modulus

**Figure 3:**
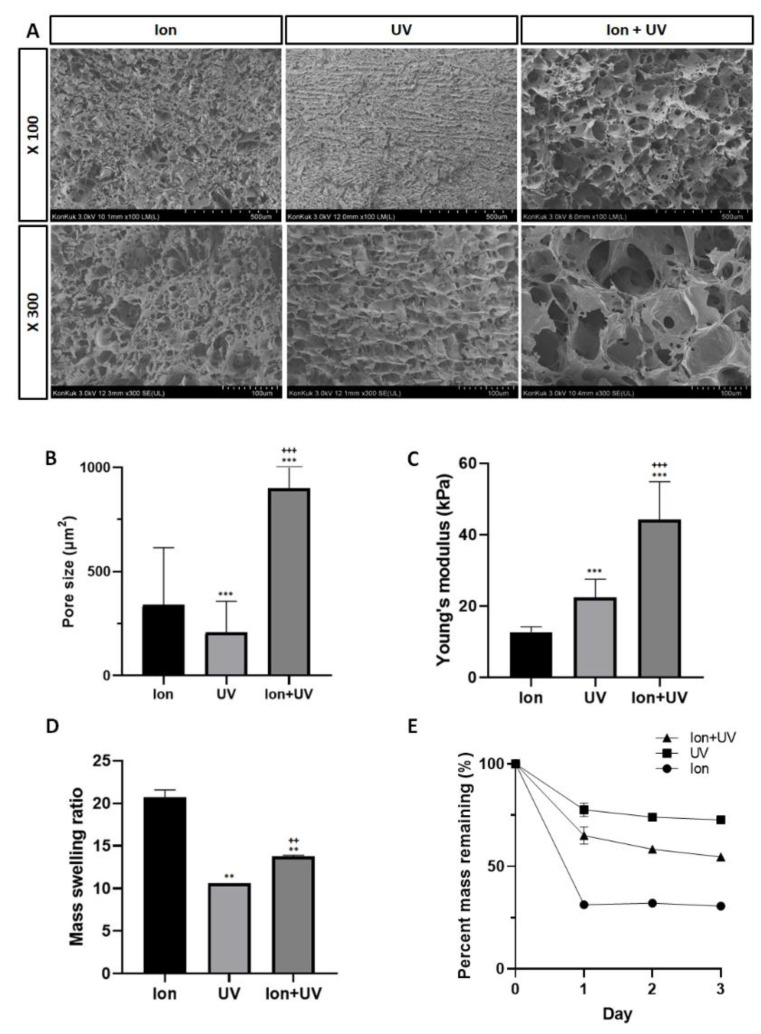
Pore distribution and mechanical analysis. (**A**) Scanning electron microscopy images of bioink prepolymer solutions cross-linked under three different conditions (ion, UV, and ion + UV). (**B**) Pore size analysis of bioink cross-linked under three different conditions (ion, UV, and ion + UV). The average pore size value for each cross-linking condition was 339.29 ± 275.01, 207.4 ± 149.09, and 897.74 ± 365.84 μm^2^ for ion, UV, and ion + UV, respectively (*n* = 8). (**C**) Young’s modulus of cross-linked bioink. Average young’s modulus for each cross-linking condition was 12.75 ± 1.35, 22.43 ± 4.71, and 44.19 ± 9.76 kPa for ion, UV, and ion + UV, respectively (*n* = 6). (**D**) Mass swelling ratio (*n* = 2) and (**E**) Percent mass remaining (%) of bioink prepolymer solutions cross-linked under three different conditions (ion, UV, and ion + UV) (*n* = 3). (*, ^+^ indicates vs. ion and UV. *** *p* < 0.001, ** *p* < 0.01, ^+++^ *p* < 0.001, ^++^ *p* < 0.01).

In has been confirmed that the pore structure of bioink allowed the sufficient space for the encapsulated cells to reside and also provided a microenvironment for supplying ample amounts of nutrients and oxygen necessary for the cells [44]. As shown in Figure 3A, the morphology of bioink prepolymer cross-linked under three conditions (ion, UV, and ion + UV) were confirmed using the field-emission scanning electron microscope (FE-SEM), and the cross-sectional morphology of the observed specimens showed a porous network. Through the pore size measurement, it was found that the prepolymer sample cross-linked with ion only appeared to have an irregular size distribution (339.29 ± 275.01 μm^2^), and the sample cross-linked by UV only showed the pore size distribution with the smallest numbers (207.4 ± 149.09 μm^2^). In the case of the bioink prepolymer cross-linked by ion + UV, the average pore size (897.74 ± 365.84 μm^2^) appeared to be the widest compared with other conditions (Figure 3B). In general, the UV cross-linking introduces more condensed pore network, and thus result in smaller average pore size distribution [45]. Therefore, when UV cross-linking is performed on ion cross-linked bioink, condensed polymers networks are formed, and the pore size becomes smaller. However, in the case of bioink with CMC and XG, the elevated viscosity interferes with polymer chain mobility of GelMA, the light-responsive moiety of bioink.

Young’s modulus was measured to assess the mechanical properties of the cross-linked bioink prepolymer under three different conditions (ion, UV, and ion + UV). It has been reported that the mechanical properties of biomaterial is one of the crucial aspects to be considered when designing a material as they affect cell proliferation, function, and differentiation [46]. As shown in Figure 3C, bioink cross-linked by double network system (ion + UV) resulted in higher Young’s modulus (44.19 ± 9.76 kPa) than a single network system formed by ion or UV cross-linking (12.75 ± 1.35 and 22.43 ± 4.71 kPa) [47].

### 3.3. Swelling and Degradation Characteristics

The swelling properties of the hydrogel change depending on the interaction between solvent vs. polymers and the structural property of polymers [48]. Moreover, they have a significant impact on the surface structure, solvent diffusion, and mechanical properties [49,50]. In Figure 3D, the mass swelling ratio, which is the expansion rate of swollen hydrogels vs. lyophilized hydrogels, is summarized according to cross-linking type (ion, UV, and ion + UV). On average, the mass swelling ratio for the bioink prepolymer cross-linked by ion was 20.7, for UV condition was 10.61, and for ion + UV condition was 13.75.

Furthermore, temporal degradation profile of ECM have a significant effect on cell migration and tissue remodeling [51] as the cells breakdown adjacent polymer network and replace them with newly produced ECM component [52,53,54,55,56]. To determine the degradation behavior, the test was performed up to day 3 without media change using DMEM supplemented with 10% FBS and 1X P/S (Figure 3E). On the first day, the bioink prepolymer cross-linked by ion only showed steep degradation profile down to 31.3% followed by steady degradation with 30.6% remaining by day 3 [57]. As expected, the samples cross-linked under UV conditions displayed the least degradation with percent mass remaining with 77.7% on day 1, and the weight remained constant throughout the test period with 74% and 72.67% on day 2 and day 3, respectively. In the case of ion + UV condition, remaining mass was 65% on day 1, 58.4% on day 2, and 54.7% on day 3. Bioink prepolymer cross-linked by ion performed without replacing DMEM was mostly decomposed on the first day, hydrogel cross-linked by UV was decomposed slowly compared to the others, whereas hydrogels cross-linked by ion + UV were decomposed gradually over time.

### 3.4. Biocompatibility Analysis

#### 3.4.1. Live/Dead Assay

**Figure 4:**
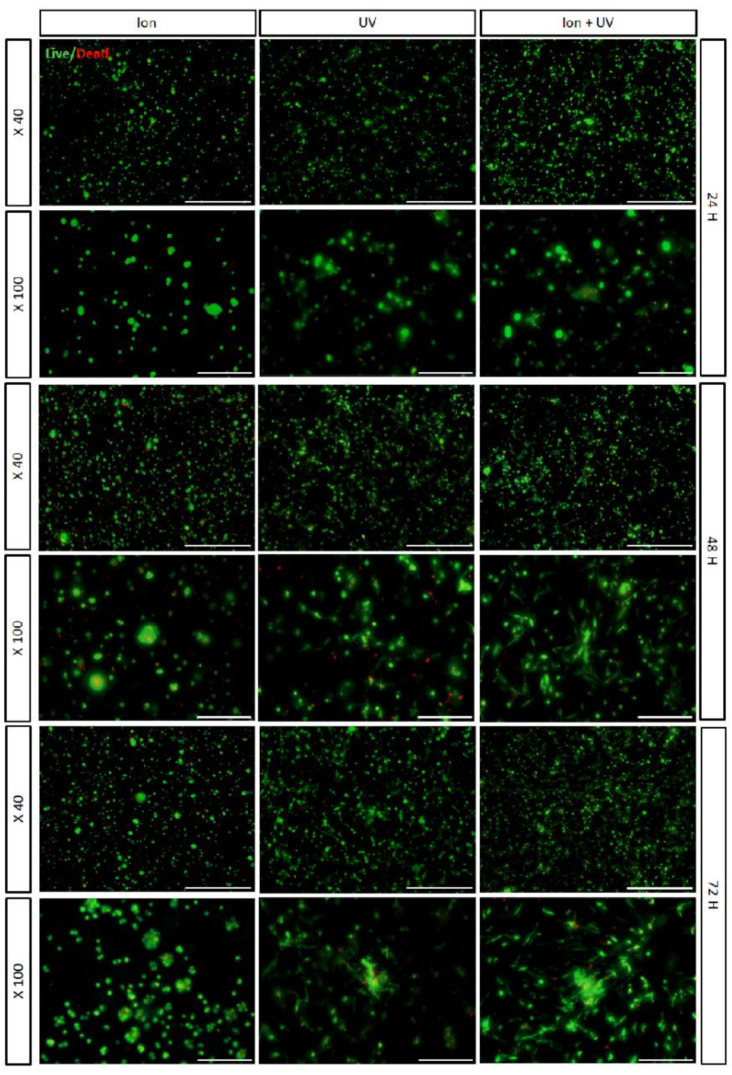
Fluorescence micrograph of Live/Dead assay. Large number of encapsulated hMSCs aggregates can be observed for ion conditions at 24, 48, and 72 h (scale bar = 40×, 500 μm; 100×, 200 μm).

To analyze the encapsulated cell behavior within the cross-linked bioink, encapsulated hMSCs were cultured for 3 days. First, Live/Dead assay was performed at 24, 48, and 72 h to visually confirm the viability of cells in the case of bioink prepolymer cross-linked under three conditions (ion, UV, and ion + UV) (Figure 4). According to the acquired LIVE/DEAD images, under ion cross-linked conditions, many cell aggregates were present. As unmodified alginate lacks cell adhesivity and therefore could not specifically interact with mammalian cells [33], the cells were aggregated in ion cross-linking conditions. In the conditions cross-linked by UV, fewer number of aggregates were present with a greater number of cells with stretched orientation. Cells encapsulated in dual cross-linked bioink (ion + UV) promoted infiltration and thus induced stretched morphology. These results can be related to difference in pore size and distribution that has been adjusted by cross-linking mechanism, which may affect cell infiltration behavior and proliferation [58]. Therefore, subsequent analysis such as proliferation, F-actin cytoskeleton, and MMP expression analysis were carried out.

#### 3.4.2. Cell Viability Analysis and Proliferation Assay

**Figure 5:**
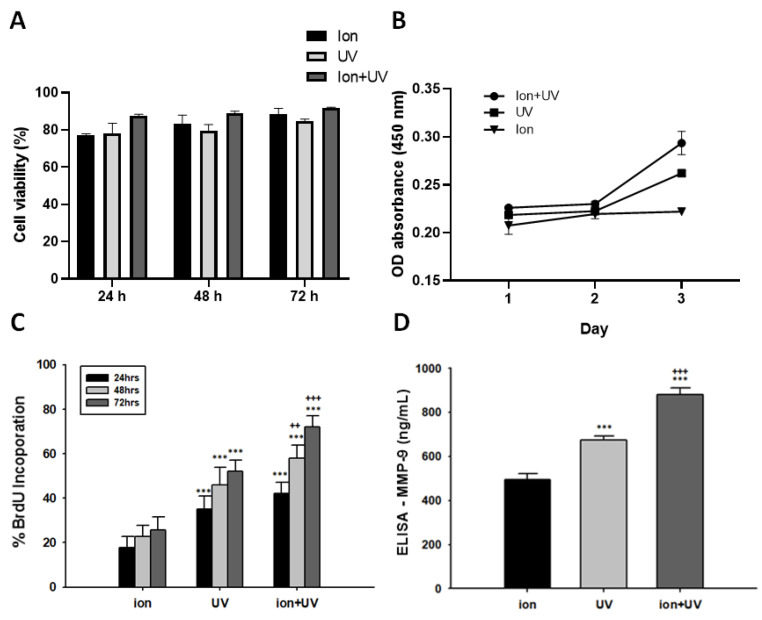
Cell viability analysis. (**A**) Cell viability assay of 3D-encapsulated hMSCs in hydrogel (*n* = 3). Cells under ion + UV condition shows better viability than other conditions. (**B**) CCK-8 cell proliferation assay (*n* = 3). The OD absorbance was measured at 450 nm. (**C**) Under ion + UV condition, compared other conditions, the BrdU incorporation were significantly higher values (*n* = 3). Data were expressed as mean ± S.D. *** indicates *p* < 0.001 vs. ion, ^++^ *p* < 0.01, and ^+++^ *p* < 0.001 vs. UV). (**D**) ELISA assay of MMP-9. The expression level of MMP-9 was highest under ion + UV condition, followed by UV condition, and lowest under ion condition (*n* = 3) (data were expressed as mean ± S.D. *** indicates *p* < 0.001 vs. ion, ^+++^ *p* < 0.001 vs. UV).

To confirm the degree of proliferation of the encapsulated hMSCs, cell viability and CCK-8 assay was performed (Figure 5A,B). To quantitatively analyze live and dead cells using the images shown in Figure 4, the ratio of live vs. total cells were calculated. Additionally, BrdU assay was also performed to confirm the proliferation of 3D-encapsulated hMSCs (Figure 5C). For cell viability (%), hMSCs showed high cell viability for the entire culture period without any significant differences between different cross-linking conditions. The cell proliferation ability of encapsulated cells was higher for ion + UV condition compared to ion and UV conditions (Figure 5B). It was also confirmed that the cell proliferation rate over time was the highest for ion + UV condition at 24, 48, and 72 h (Figure 5C). Lastly, ELISA analysis of MMP-9 was performed to measure the matrix metalloproteinase-9 (MMP-9) expression level, which degrades ECM such as gelatin and collagen (Figure 5D) [59]. As a result, the expression of MMP-9 was the highest in the hydrogel cross-linked with ion + UV condition.

#### 3.4.3. Phalloidin/DAPI Staining Assay

**Figure 6:**
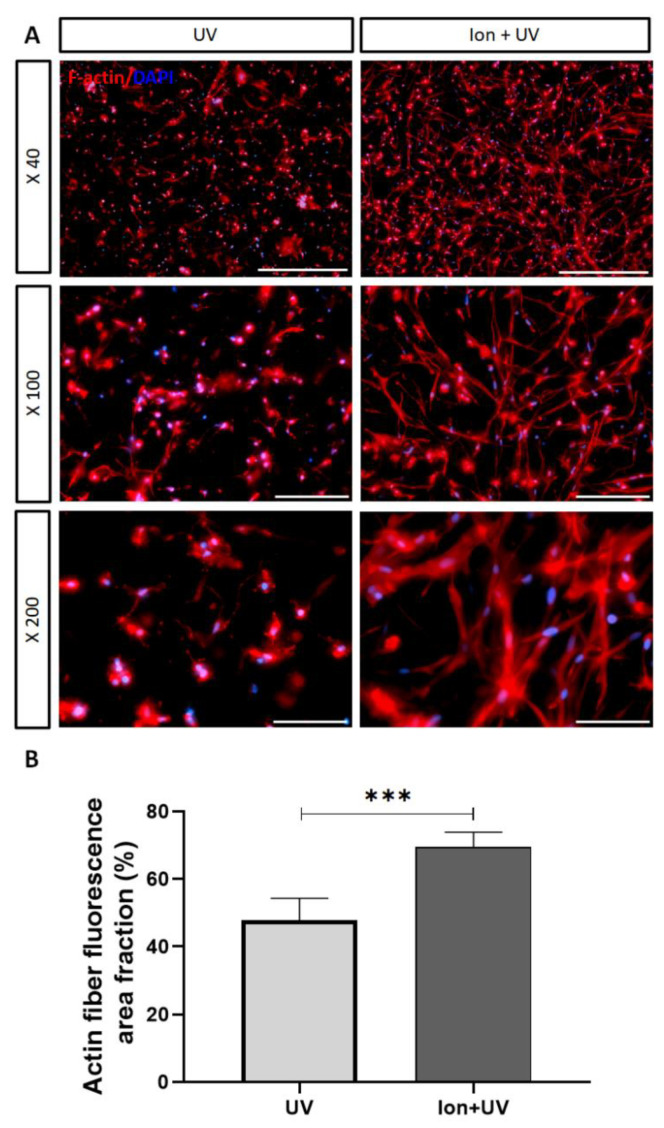
Fluorescence micrograph of phalloidin/DAPI staining assay. (**A**) Cells were stained with phalloidin (red) and DAPI (blue) at 72 h (scale bar = 40×, 500 μm; 100×, 200 μm; 200×, 100 μm). (**B**) Percent of actin fiber fluorescence area fraction (*n* = 5) (data were expressed as mean ± S.D. *** *p* < 0.001).

Phalloidin/DAPI staining assay was performed to confirm the filamentous actin and nuclei of cells. As shown in Figure 6A, filamentous actin was not stretched out long in the bioink prepolymer cross-linked by UV only showing incomplete connection between the encapsulated hMSCs. On the other hand, in the case of ion + UV cross-linked prepolymer samples, great number of long stretches of filamentous actin were present as thick bundles with sufficient connection between the encapsulated cells. The reason for these results can be related with Figure 2 as the low average pore size of UV cross-linked bioink does not provide space for filamentous actin to stretch and interconnect. On the other hand, hydrogel cross-linked with ion + UV has a wider pore size for the filamentous actin to stretch out. For further analysis, actin fiber fluorescence area fractioning was performed to quantify the area of filamentous actin relative to the total area (Figure 6B). According to the results, it was confirmed that the ion + UV sample ensured larger areas of filamentous actin compared to the hydrogel cross-linked with UV.

#### 3.4.4. Immunocytochemistry Staining Assay

**Figure 7:**
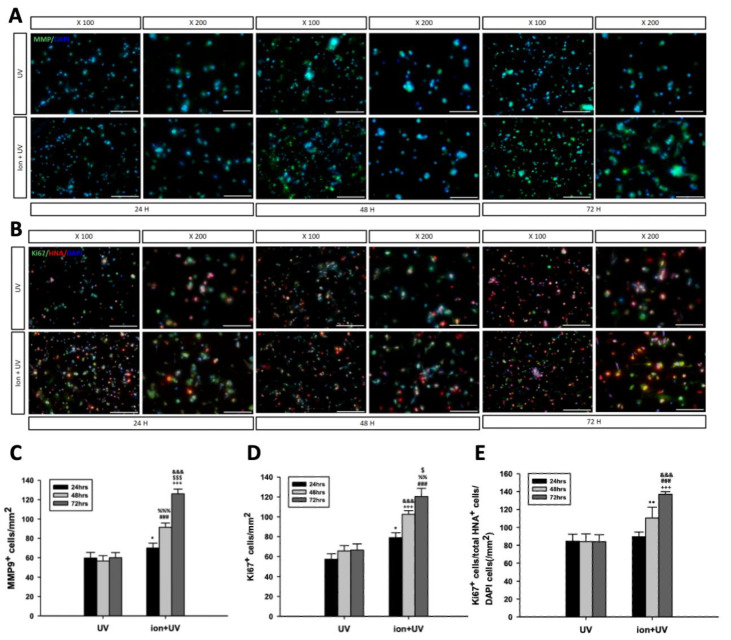
Immunocytochemistry staining assay. (**A**) Fluorescence images showing MMP-9/DAPI staining assay. (**B**) Fluorescence micrograph of Ki-67/HNA/DAPI staining assay (scale bar = 100×; 200 μm; 200×, 100 μm). (**C**) MMP-9-positive cells/mm^2^ (data were expressed as mean ± S.D. * indicates *p* < 0.05 vs. 24 h UV, ^###^ *p* < 0.001 vs. 48 h UV, ^+++^ *p* < 0.001 vs. 72 h UV, ^%%%^ *p* < 0.001 and ^$$$^ *p* < 0.01 vs. 24 h ion + UV, ^&&&^ *p* < 0.001 vs. 48 h ion + UV). (**D**) Ki-67-positive cells/total HNA-positive cells/DAPI cells (data were expressed as mean ± S.D. * indicates *p* < 0.05 vs. 24 h UV, ^+++^ *p* < 0.001 vs. 48 h UV, ^###^ *p* < 0.001 vs. 72 h UV, ^&&&^ *p* < 0.001 and ^%%^ *p* < 0.01 vs. 24 h ion + UV, ^$^ *p* < 0.05 vs. 48 h ion + UV). (**E**) Ki-67-positive cells/mm^2^ (data were expressed as mean ± S.D. ** indicates *p* < 0.01 vs. 48 h UV, ^+++^ *p* < 0.001 vs. 72 h UV, ^###^ *p* < 0.001 vs. 24 h ion + UV, ^&&&^ *p* < 0.001 vs. 48 h ion + UV). All experiments were conducted at least 3 times.

Immunocytochemistry assay for MMP-9/DAPI was performed to visually confirm the expression of MMP-9 of the encapsulated (Figure 7A). MMP-9 in hydrogel plays an important role in the process of decomposing ECM, creates spaces for cells to grow and remodel surrounding tissues for tissue regeneration, and even takes part in angiogenesis [60]. After 72 h, it was found that MMP-9 was expressed in both conditions (ion and ion + UV). The ratio of expressed MMP-9 and DAPI was calculated based on the results measured from the MMP-9/DAPI staining assay and is depicted in Figure 7C. Subsequently, a significantly higher degree of MMP-9 expression was confirmed in the bioink prepolymer cross-linked under ion + UV conditions.

Ki-67/HNA/DAPI staining assay was also performed to visually confirm the expression of Ki-67 in 3D-encapsulated cells (Figure 7B). Ki-67 is known to be expressed in the proliferative phase rather than at the stationary phase in the cell cycle and is used as a proliferation marker. Human-specific HNA marker was used to identify antigen associated with the nuclei of encapsulated hMSCs. First, the expression of Ki-67 per total area was measured using the stained data (Figure 7D). According to the calculation, it was confirmed that the bioink prepolymer cross-linked with ion + UV showed higher expression rate. Statistical values under ion + UV conditions were higher for all the time points at 24, 48, and 72 h. In order to confirm whether the Ki-67 was expressed in hMSCs, the ratio of HNA vs. Ki-67-stained cells to DAPI-stained cells was confirmed (Figure 7E). Since the statistical values for Figure 7D, E showed similar patterns, it was confirmed that Ki-67 expression is derived from proliferating hMSCs.

#### 3.5. 3D Bioprinted hMSCS

**Figure 8:**
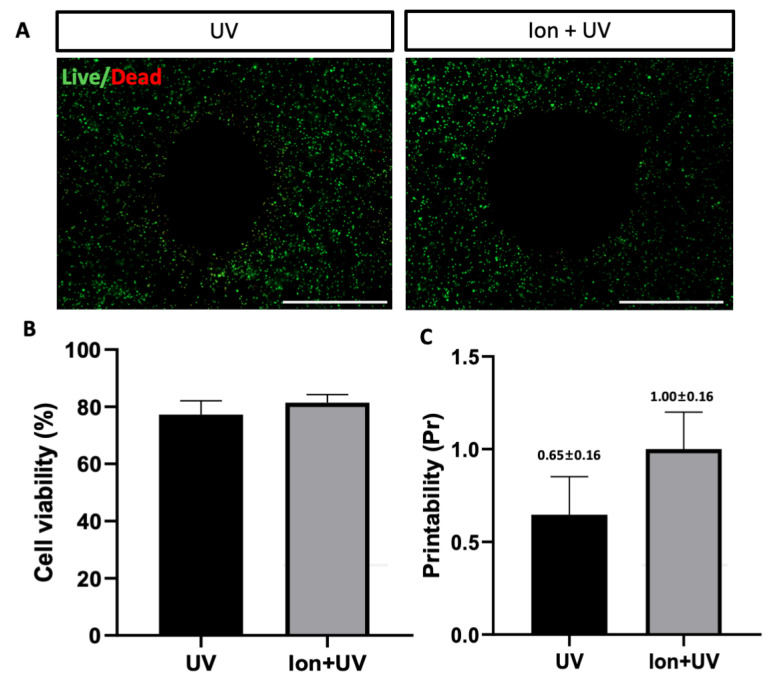
Cell viability of 3D-bioprinted hMSCs. (**A**) Fluorescence micrograph of Live/Dead assay for printed fiber. Bioink composition was 10:2:2:2 (GelMA:alginate:CMC:XG) and cross-linking conditions were UV and ion + UV. (**B**) Cell viability results of 3D-encapsulated cell in printed fiber (UV vs. ion + UV). (**C**) Printability (Pr) of printed fiber after 24 h (scale bar = 100×, 200 μm).

The printing of encapsulated cells was performed using a 3D extrusion-based printer. The printed cells were cultured for 24 h, followed by a live/dead assay for observation (Figure 8A). To compare the printability of bioink, UV and ion + UV were used as cross-linking conditions. When the cell viability under UV and ion + UV conditions was compared, both conditions showed good cell viability (%) as can be seen in Figure 8B. After 24 h of incubation, the maintaining printed structure was also confirmed through an experiment (Figure 8C). It was confirmed that the printed structure was maintained as it was under ion + UV condition, whereas the structure cross-linked under the UV condition showed that the Pr value decreased compared to the initial printed structure (Figure 2B). This means that the structure cross-linked under ion + UV conditions was superior to other conditions in terms of cell viability and shape maintenance.

## 4. Conclusions

Bioink, which consists of cells and polymeric materials, should have characteristics that can regulate printability, gelation, degradation, and biocompatibility. However, the fact that printability and biocompatibility do not increase at the same time poses a problem. To solve this problem, we developed a bioink with enhanced printability by utilizing CMC and XG and increased biocompatibility through the introduction of increased average pore size of cross-linked bioink. The printability and rheological analysis showed that the bioink has improved mechanical properties with increased average pore size at the same time. As a result, it was confirmed that the printability could be adjusted by controlling the CMC and XG ratio. Cell viability and staining analyses were also performed up to day 3 to evaluate biocompatibility. When the cells were observed via the staining assay, it was confirmed that the morphology of the encapsulated cells cross-linked under the ion + UV condition showed higher number of filamentous actin activity as well as proliferation. Through the cell viability analysis, it was confirmed that the bioink prepolymer cross-linked under the ion + UV condition showed significantly higher cell viability and proliferation. As a result, using the bioink formulation introduced in this study, it is possible to adjust its printability to suit printing purposes with improved biocompatibility. These results suggest that the bioink proposed in this paper can be a useful option in various fields utilizing extrusion-based 3D bioprinting and that this will be a convenient method for more appropriate use of bioink.

## Data Availability

The authors do not wish to share the data on public.
